# L‐Carnitine metabolism, protein turnover and energy expenditure in supplemented and exercised Labrador Retrievers

**DOI:** 10.1111/jpn.13391

**Published:** 2020-06-17

**Authors:** Jessica Lyn Varney, Jason William Fowler, Trenda Clarice McClaughry, Karen Vignale, Justina Caldas, Jordan Taylor Weil, Craig Nelson Coon

**Affiliations:** ^1^ Four Rivers Kennel LLC Walker MO USA; ^2^ ELIAS Animal Health LLC Olathe KS USA; ^3^ Kemin Industries Inc. Des Moines IA USA; ^4^ Cobb‐Vantress Inc. Siloam Springs AR USA; ^5^ University of Arkansas Fayetteville AR USA

**Keywords:** dog, energy expenditure, Labrador Retriever, L‐carnitine, protein turnover

## Abstract

L‐Carnitine is critical for protection against bioaccumulation, long‐chain fatty acid transportation and energy production. Energy production becomes important as the body maintains lean mass, repairs muscles and recovers from oxidative stress. The aim was to investigate the effects of supplemented L‐carnitine on protein turnover (PT), energy expenditure (EE) and carnitine metabolism in muscle/serum of Labrador Retrievers. In a series of experiments, all dogs were fed a low‐carnitine diet and sorted into one of two groups: L‐carnitine (LC) supplemented daily with 125 mg L‐carnitine and 3.75 g sucrose or placebo (P) supplemented with 4 g sucrose daily. The experiments consisted of analyses of muscle/serum for L‐carnitine content (EXP1), a protein turnover experiment (EXP2) and analysis of substrate utilization via indirect calorimetry (EXP3). EXP1: 20 Labradors (10 M/10 F) performed a 13 week running regimen. L‐Carnitine content was analysed in the serum and *biceps femoris* muscle before/after a 24.1 km run. LC serum had higher total (*p* < .001; *p* = .001), free (*p* < .001; *p* = .001) and esterified (*p* = .001; *p* = .003) L‐carnitine pre‐ and post‐run respectively. LC muscle had significantly higher free L‐carnitine post‐run (*p* = .034). EXP2: 26 Labs (13 M/13 F) performed a 60‐day running regimen. For the final run, half of the Labradors from each treatment rested and half ran 24.1 km. Twenty‐four Labradors received isotope infusion, and then, a biopsy of the biceps femoris of all 26 Labradors was taken to determine PT. Resting/exercised LC had a lower fractional breakdown rate (FBR) versus P group (*p* = .042). LC females had a lower FBR v. P females (*p* = .046). EXP3: Respiration of 16 Labradors (8 M/8 F) was measured via indirect calorimetry over 15 week. All dogs ran on a treadmill for 30 min at 30% VO_2_ max (6.5 kph), resulting in higher maximum and mean EE in LC females v. P females (*p* = .021; *p* = .035). Implications for theory, practice and future research are discussed.

## 1 INTRODUCTION

L‐Carnitine has gained attention in recent years regarding its purported beneficial effects as a supplement in exercise performance and recovery. Although many research studies have examined the benefit of supplemented L‐carnitine in humans and other animals, few studies have been performed on L‐carnitine's impact on canine exercise performance and recovery. Studies performed on the supplementation of L‐carnitine in sled dogs (Iben, 1998) and in beagles during exercise (Iben, 1999) found no effect on heart rate or blood parameters such as serum lactate, creatine kinase and haematocrit. Past studies have shown a positive influence on blood parameters such as serum lactate, creatine kinase and aspartate aminotransferase; however, L‐carnitine was provided to test subjects in a mixture of performance‐enhancing supplements, making it difficult to attribute the results solely to L‐carnitine (Grandjean & Fuks, 1993; Huntingford, Kirn, Cramer, Mann, & Wakshlag, 2014).

L‐Carnitine supplementation has been shown to mediate effects of tissue degradation, muscle hypoxia and free radical formation after squat exercise in humans (Volek et al., 2001). L‐Carnitine has also been thought to impact substrate availability, oxidation and energy metabolism during endurance exercise in humans (Lohninger et al., 2005). Epp, Erickson, Woodworth, and Poole (2007) found that L‐carnitine supplementation increased the potential for oxygen transport in racing greyhounds, while reducing indicators of muscle damage. Previous studies reported 125 mg L‐carnitine provided daily to Labrador Retrievers produced a positive effect on activity intensity, body composition, muscle recovery and oxidative capacity during a 14‐week feeding/exercise regimen (Varney, Fowler, Gilbert, & Coon, 2017).

The major objectives of the present studies were (a) to quantify L‐carnitine deposition in both blood and muscle following a 14‐week feeding period, (b) to evaluate the effect of L‐carnitine supplementation on protein synthesis and breakdown via a protein turnover (PT) experiment and (c) to determine whether L‐carnitine supplementation increases the energy expenditure (EE) and oxygen consumption in Labrador Retrievers.

## 2 MATERIALS AND METHODS

All animal care and procedures for Protocol FRK‐04 were reviewed and approved by the Institutional Animal Care and Use Committee at Four Rivers Kennel, LLC.

### 2.1 Animals and housing

**FIGURE 1:**
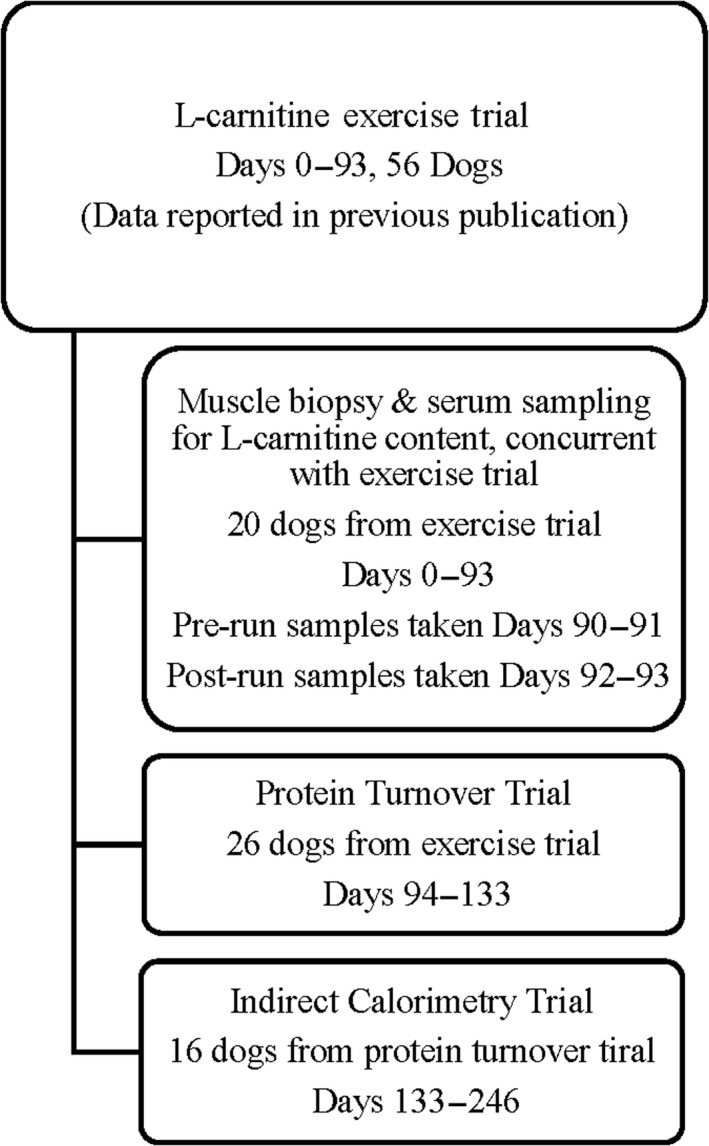
Outline and supplementation length of dogs used in all three trials

All Labrador Retrievers used in each of the described experiments were intact and ranged from 1 to 4 years of age. Muscle biopsies and serum samples were taken from 20 Labradors (10 M/10 F) for L‐carnitine content quantification (EXP1) as part of a 14‐week endurance exercise trial, which used 56 dogs total (Varney et al., 2017). Twenty‐six Labradors (13 M/13 F) from the previous exercise trial were used for the protein turnover experiment (EXP2) and 16 Labradors (8 M/8 F) from the PT experiment were used in a calorimetry experiment (EXP3). Dogs remained on their respective treatments during all three phases (Figure 1). All dogs were housed in individual kennels overnight and allowed free access to outside airing yards for 6–8 hr daily, weather permitting. All dogs had ad libitum access to automatic waterers at all times. The dogs were fed once daily in the morning as per their treatment requirements. Prophylactic heartworm (HEARTGARD Plus [ivermectin/pyrantel]; Merial) and parasite prevention (NexGard [afoxlaner]; Merial) were administered monthly.

### 2.2 Diets

A low‐L‐carnitine basal diet was formulated by Dr. George Collings (Collings Nutritional Solutions) and prepared in the extrusion facilities of Kansas State University, Manhattan, KS. Feed from the same batch was used for all three studies. The nutrient content of the test diet was determined prior to the start of the studies (Table 1). The metabolizable energy (ME) for the basal diet was determined using the indicator method (AAFCO Official Publication, 2015) and was analysed for crude protein and gross energy via bomb calorimetry (University of Arkansas Central Analytical Laboratory). L‐Carnitine levels in the diet were tested using a radioisotopic enzymatic method (Parvin & Pande, 1977) (Metabolic Analysis Labs). Feed consumption was determined daily by weighing feed provided and feed refusals. Each female dog was offered 600 g daily, and each male dog was offered 800 g daily.

**TABLE 1:** Ingredient composition and analysed nutrient content of the low L‐carnitine basal diet

Ingredient	Percentage	Nutrient	Percentage
Corn, ground	42.86	Dry matter	92.0
Chicken meal	29.00	Moisture	8.0
Wheat, ground	12.80	Crude Protein	27.4
Rice, brewer's	5.50	Crude fat	12.8
Beet pulp	5.50	Crude fiber	2.57
Egg, dried	1.11	Calcium	1.26
Flaxseed	1.11	Phosphorus	0.89
Salt, plain	0.59	Ash	6.04
Potassium chloride	0.55	Methionine	0.54
Mixed tocopherols	0.22	Lysine	0.98
L‐Lysine	0.22	Sodium	0.33
DL‐Methionine	0.20	Potassium	0.64
2011‐No K‐CNS vitamin premix	0.13	Magnesium	0.12
2011–01 CNS mineral premix	0.11	Iron	263.45
Choline chloride 60%	0.11	Copper ppm	20.94
*Analysed*		Zinc ppm	233.44
L‐Carnitine (ppm)	19.3	Linoleic Acid	3.82
Metabolizable energy (kcal/kg)	3,988	Omega 6 Fatty Acids	3.51

For the PT study, all dogs were fed a non‐meat diet (analysed for the absence of 3‐methylhistidine [3MH]; Table 2) with added supplements for 3 days prior to isotopic infusion to ensure the 3MH found in the blood represented the skeletal muscle protein breakdown rather than from the diet.

**TABLE 2:** Ingredient composition and analysed nutrient content of the 3MH free diet for protein turnover

Ingredient	Percentage	Nutrient	Percentage
Whey protein concentrate	14.22	Dry matter	90.00
Ground corn	13.04	Moisture	10.00
Ground wheat	12.80	Crude Protein	26.50
Dried whole egg	10.24	Crude fat	15.00
Dehulled soybean meal	10.00	Crude fibre	2.80
Dried fat (Ho‐Milc 7–60)	10.00	Nitrogen free Extract	37.50
Dried beet pulp	6.00	Calcium	1.40
Brewers dried yeast	5.21	Phosphorus	0.80
Ground flaxseed	4.32	Ash	7.20
Soybean oil	4.10	Sodium	0.38
Bacon flavour	3.00	Potassium	0.85
Dicalcium phosphate	2.32	Magnesium	0.15
Calcium carbonate	1.92	Iron ppm	361
Salt	0.67	Copper ppm	14.00
Potassium chloride	0.55	Zinc ppm	250
Vitamin/Mineral premix	0.36	*Analysed*	
L‐Lysine	0.22	3MH %	0
DL‐Methionine	0.20	Metabolizable Energy (kcal/kg)	4,000

### 2.3 Added supplements

For the duration of all experiments, the L‐carnitine group was orally supplemented daily with 3.75 g sucrose and 250 mg Carniking brand L‐carnitine powder (Lonza Group Ltd), providing 125 mg L‐carnitine. The placebo group was solely supplemented with 4 g sucrose daily. Dry supplements were made in stock and measured individually each day. The prepared supplements were added to the top of 200 g of basal food to ensure each dog received the full dose of supplements. After each dog consumed the 200 g of basal kibble with dry supplements, dogs were then given the rest of their meal.

All dogs used for the muscle/serum L‐carnitine analysis had been on their respective treatments for 93 days prior to analysis. Pre‐run samples were taken at Day 90 of supplementation for the females and Day 91 for the males, while the post‐run samples for females were taken at Day 91 and Day 92 for the males. The muscle/serum L‐carnitine content analysis was derived from a larger experiment of 56 dogs, with 26 dogs from this continuing to the PT experiment. The PT experiment started at 93 days and ended at 133 days of supplementation. Sixteen dogs from the PT experiment continued to the calorimetry experiment, which started at 133 days and ended at 246 days of supplementation (Figure 1).

### 2.4 Stable isotope infusion and sampling

During the PT experiment, 24 dogs were infused via cephalic intravenous catheter with a 15N phenylalanine 2% solution (Cambridge Isotope Laboratories) over 10 min, providing 70 mg phenylalanine per kilogram of body weight. Forty‐five minutes after completing the 15N phenylalanine infusion, a veterinarian performed a biopsy of the biceps femoris muscle with a 5‐mm Bergstrom muscle biopsy needle. Muscle biopsies were also taken from two control dogs that were fed the basal diet. Dogs in the control group were not infused in order to provide a background sample and determine the natural amount of 15N phenylalanine in the muscle (data not included in results). Samples were immediately frozen in liquid nitrogen and stored at −80°C until analysis. In conjunction with the muscle biopsies, a blood sample was collected from each dog via jugular venipuncture and centrifuged at 1,300 **g** × 15‐min. Serum was aliquoted into polypropylene tubes and stored at −80°C until analysis.

### 2.5 Sample processing

The acid‐soluble fraction containing free amino acids were removed by addition of 2% (w/v) perchloric acid. After homogenization, samples were centrifuged at 3,000 **g** and the supernatant containing free amino acids removed. The protein precipitate was washed three times with 2% perchloric acid before being hydrolysed in 6N hydrochloric acid. The supernatant and precipitate, respectively, were then run through an ion‐exchange column packed with Dowex 50WX8‐200. Phenylalanine and 3MH were eluted with 2 ml of 4N ammonium hydroxide and 1 ml of nanopure H_2_O into a new vial and dried under vacuum. The tert‐butyldimethylsilyl (tBDMS) derivative was formed by addition of 800 μl of C2CH3CN‐MTBSTFA (acetonitrile‐N‐methyl‐N‐tert‐butyldimethylsilyl)trifluoroacetamide) (1:1) and incubated at 110°C for 60 min.

### 2.6 GC/MS analysis

Analysis of the protein precipitate of muscle samples and free amino acids was carried out on an Agilent 7890A GC system attached to an Agilent 5975C mass spectrometer. Helium was used as the carrier gas at 1 ml/min; a 1 µl volume was injected in splitless mode. Starting oven temperature was 150°C and increased 50°C/min to 200°C, after which temperature was increased 20°C/min to 270°C and was held for 5.5 min. The mass spectrometer was operated under electron ionization and selective ion monitoring mode. The 394 and 395 m/z fragments, representing the M and M + 1 fragments of phenylalanine, were monitored.

3MH was determined on the same mass spectrometer. Helium was used as the carrier gas at 1 ml/min; a 1 µl volume was injected in splitless mode. Starting oven temperature was 110°C and held for 0.65 min, and then, temperature was increased 30°C/min to 250°C and held for 10 min. The mass spectrometer was also operated under electron ionization and selective ion monitoring modes. The 238 m/z fragment of 3MH was monitored. The fractional synthesis rate (FSR) was calculated as follows: ks = APEb/APEf × 1/t × 100, where APEb, = 15N atom per cent excess (relative to natural abundance) of phenylalanine in protein; APEf = 15N atom per cent excess of free phenylalanine in tissues, assumed as the precursor pool; and *t* = time [d]. The fractional breakdown rate (FBR) was calculated as follows: kd = 3MH daily excretion/3MH muscle pool × 100.

### 2.7 Calorimetry evaluation

The system used to acquire volume of oxygen consumption and EE (heat production) from the dogs was an open‐circuit indirect calorimetry machine (Columbus Instruments) that employs the air from the surrounding environment (Pouteau et al., 2002), which was calibrated daily. To recover the gas sample, a specially designed face mask was used. A small sample drawn for gas analysis was dried to ensure that the readings were made in a sample that were not under the influence of water vapour air exiting the chamber. O_2_ and CO_2_ sensors were connected to software and instant values of O_2_ and CO_2_ measured. EE was calculated with the formula for dogs: EE (Kcal/d) = 3.94 VO_2_ + 1.11 VCO_2_ (Cunningham, 1990).

### 2.8 Experiment 1: Muscle biopsy and serum sampling

Twenty dogs (10 M/10 F) were sorted into two treatment groups that were equalized between gender, genetics (related dogs) and body composition. The L‐carnitine group was supplemented with 125 mg L‐carnitine powder and 3.75 g sucrose each day and the placebo group supplemented with 4 g sucrose each day. All dogs were fed a low‐carnitine basal diet in which the daily amounts offered were determined based on maintaining each dog's initial starting body weight. Feed offered and refused was weighed daily to measure consumption. All dogs performed a running exercise regimen during the study. The exercise regimen included two long endurance runs per week increasing incrementally from 8.8 to 16.1 km, with a final long run of 24.2 km. To evaluate the levels of total L‐carnitine, free L‐carnitine and L‐carnitine esters in the muscle and blood, muscle biopsies and serum samples were collected from 10 dogs in each treatment group at 24 hr before and 1 hr after the final run. Muscle biopsies were collected by a veterinarian from the biceps femoris using a 5‐mm Bergström biopsy needle while the dogs were sedated for dual‐energy X‐ray absorptiometry (DEXA) body composition scans. Blood serum was collected via jugular venipuncture. All samples were frozen at −80°C and shipped to Metabolic Analysis Labs for analysis.

### 2.9 Experiment 2: Protein turnover

Protein turnover was determined in 26 dogs (13 M/13 F) after a 60 days exercise study. Twelve dogs were fed the basal diet along with 125 mg L‐carnitine and 3.75 g sucrose (L‐carnitine group), and 14 dogs were fed the basal diet and 4 g sucrose placebo only (Placebo group). Two dogs from the placebo group were not enriched with isotopes and did not perform any running exercise, in order to measure the natural amount of 15N phenylalanine in the muscle and serum for background purposes. All dogs completed a running exercise programme, running freely alongside an all‐terrain vehicle in the bush, over the course of 60 days. Two endurance runs were performed each week, one at a steady pace and the other interspersed with 100 m fartleks (periods of fast running intermixed with periods of slower running). The runs began at 8 km and increased incrementally to 16 km over 8 weeks. On day 60, half of the dogs from each treatment group ran a final 24 km. The other half of the dogs from each treatment group did not run and served as a resting comparison. Sixty minutes after the final long run when heart rate and body temperature were normal, the two treatment groups were infused with a bolus dose of 15N phenylalanine and serum and muscle biopsy samples were taken.

### 2.10 Experiment 3: Oxygen consumption and energy expenditure

Oxygen consumption (VO_2_) and EE were determined during a 15‐week treadmill training and running regimen. Sixteen dogs (8 M/8 F) were sorted into two treatment groups: eight dogs in the L‐carnitine group (4 M/4 F) were fed the basal diet and supplemented daily with 125 mg L‐carnitine and 3.75 g sucrose, and eight dogs (4 M/4 F) in the placebo group were fed the basal diet and supplemented daily with 4 g sucrose (P group). Both groups were trained to run on a high‐performance treadmill (Fitnex) while attached to an open‐air indirect calorimetry system. For acclimation and exercise training purposes, each dog was exercised for 30 min at incrementally increasing speeds on the treadmill twice per week for 13 weeks. During week 14, each dog was exercised for 30 min on a zero incline at a speed of 6.5 km per hour (kph) (previously determined to be 30% VO_2_ max) and VO_2_ and EE were determined. During week 15, each dog was exercised at a speed of 10.5 kph (previously determined to be 50% VO_2_ max) for 15 min and VO_2_ and EE determined. Before each speed evaluation, the dogs underwent a 5‐min warm‐up at a speed of 3.2 kph (data not recorded). All dogs were exercised on the treadmill in random order each week. Four dogs were tested per day in the morning 1 hr after consuming 200 g of feed each and prescribed supplements.

### 2.11 Body composition scans

All dogs were scanned using a GE Lunar Prodigy dual‐energy X‐ray absorptiometry machine (General Electric Company) prior to the PT experiment to ensure each group was balanced by body fat and lean mass (Table 3). All dogs were anaesthetized by a licensed veterinarian for the scans using dexmedetomidine (Dexdomitor; Zoetis Inc), torbutrol (Zoetis Inc) and atropine (Vedco Inc).

**TABLE 3:** Body composition results used to allocate dogs into equal treatment groups for the protein turnover experiment

	L‐Carnitine	*SD*	*n*	Placebo	*SD*	*n*	*p*‐Value
Total tissue mass (kg)							
Overall	27.67	4.26	12	27.93	4.71	12	.887
Male	31.11	1.91	6	32.03	2.06	6	.441
Female	24.23	2.79	6	23.83	1.54	6	.764
Fat mass (kg)							
Overall	4.12	1.28	12	5.40	1.84	12	.059
Male	3.78	1.22	6	5.40	2.45	6	.177
Female	4.47	1.32	6	5.40	1.15	6	.222
Lean mass (kg)							
Overall	24.31	4.78	12	22.61	4.57	12	.384
Male	28.33	1.10	6	26.64	2.03	6	.101
Female	20.28	3.18	6	18.58	1.74	6	.277
Body fat (%)							
Overall	15.26	5.33	12	19.68	6.62	12	.086
Male	11.97	3.23	6	16.65	7.08	6	.172
Female	18.55	5.12	6	22.70	4.90	6	.182
Bone mineral density (g/cm^2^)							
Overall	0.61	0.07	12	0.63	0.07	12	0.655
Male	0.66	0.02	6	0.67	0.05	6	0.627
Female	0.58	0.02	6	0.59	0.05	6	0.694

### 2.12 Statistical analysis

Statistical analysis was performed using JMP Pro (version 11.2.0; SAS Institute Inc.) and GraphPad Prism (version 6; Graphpad Software, Inc.). GraphPad Prism 6.0 was used to compare means between groups for muscle and serum L‐carnitine content, feed intake and body composition using unpaired *t*‐tests. For PT, JMP 11.2.0 was used to create a mixed model by treatment, sex and exercise. For indirect calorimetry, JMP 11.2.0 was used to create a mixed model by treatment, sex, speed, testing period and subject as a random variable. Results were considered significant at *p*‐value <.05. Results given are presented as mean (*SD*).

## 3 RESULTS

### 3.1 Muscle biopsies and serum sampling

Pre‐run serum levels of total L‐carnitine were significantly higher in the L‐carnitine group at 67.52 (23.45) µM/L versus the placebo group at 26.46 (8.81) µM/L (*p* < .001). Free L‐carnitine levels were also significantly higher in the L‐carnitine group at 50.20 (13.80) µM/L, compared to placebo group at 22.00 (7.03) µM/L (*p* < .001). L‐Carnitine esters were significantly higher in the L‐carnitine group at 17.32 µM/L compared to placebo group at only 4.46 µM/L (*p* < .001). L‐Carnitine group also had a significantly higher ester to free ratio (E/F ratio) at 0.33 (0.03), compared to placebo group at 0.20 (0.02; *p* = .001; Table 4).

**TABLE 4:** Serum samples analysed for L‐carnitine content, at pre‐run (1 hr prior to final long run) and post‐run (1 hr after final long run) time intervals

	L‐Carnitine *n* = 10	*SD*	Placebo *n* = 10	*SD*	*p*‐Value
Pre‐run					
Total (µM/L)	67.52	23.45	26.46	8.81	<0.001
Free (µM/L)	50.20	13.80	22.00	7.03	<0.001
Esterified (µM/L)	17.32	9.86	4.46	1.94	<0.001
E/F ratio	0.33	0.10	0.20	0.05	0.001
Post‐run					
Total (µM/L)	51.16	19.72	25.12	8.36	0.001
Free (µM/L)	31.94	12.32	15.64	5.19	0.001
Esterified (µM/L)	19.22	8.01	9.48	4.28	0.003
E/F ratio	0.61	0.11	0.63	0.21	0.727

After the final run, the L‐carnitine group had significantly higher serum levels of total L‐carnitine at 51.16 (19.72) µM/L compared to placebo at 25.12 (8.36) µM/L (*p* = .001). Free L‐carnitine levels were higher in the L‐carnitine group at 31.94 (12.32) µM/L versus placebo at 15.64 (5.19) µM/L (*p* = .001). Esterified L‐carnitine was also higher in L‐carnitine group at 19.22 (8.01) µM/L compared to placebo at 9.48 (4.28) µM/L (*p* = .003).

Muscle biopsies taken after the final long run showed the L‐carnitine group had significantly more free L‐carnitine available in the muscle after the final run compared to placebo group (*p* = .034; L‐carnitine: 4,681 (292) nmol/g v. Placebo: 3,735 (290) nmol/g; Table 5).

**TABLE 5:** Muscle (biceps femoris) biopsies analysed for L‐carnitine content. Data are presented as mean (*SD*)

	L‐Carnitine *n* = 10	*SD*	Placebo *n* = 10	*SD*	*p*‐value
Pre‐run					
Total (µM/L)	67.52	23.45	26.46	8.81	<.001
Free (µM/L)	50.20	13.80	22.00	7.03	<.001
Esterified (µM/L)	17.32	9.86	4.46	1.94	<.001
E/F ratio	0.33	0.10	0.20	0.05	.001
Post‐run					
Total (µM/L)	51.16	19.72	25.12	8.36	.001
Free (µM/L)	31.94	12.32	15.64	5.19	.001
Esterified (µM/L)	19.22	8.01	9.48	4.28	.003
E/F ratio	0.61	0.11	0.63	0.21	.727

### 3.2 Protein turnover

Average body weights of L‐carnitine group versus placebo group were not significantly different over the course of the study, with the L‐carnitine group averaging 29.09 (5.21) kg and the placebo group averaging 30.26 (4.88) kg (*p* = .211). Body composition results for assigning equalized treatment groups are reported in Table 3.

Female dogs in both groups were offered 600 g of feed each day, and male dogs were offered 800 g of feed each day for the duration of the study. Feed consumption in the L‐carnitine group averaged 620 (129) g, and the placebo group averaged 592 (112) g (*p* = .199). L‐Carnitine females averaged 502 (51) g each day, and placebo females averaged 499 (63) g (*p* = .926). L‐Carnitine males averaged 737 (28) g, while placebo males averaged 685 (52) g each day (*p* = .057).

All females had a significantly higher FBR compared to all males (*p* = .044; female: 3.70 (2.57) % vs. male: 1.93 (1.94) %). Exercised females had a significantly higher FBR at 5.14 (0.81) % compared to 2.26 (3.11) % for non‐exercised females (*p* = .021).

**FIGURE 2:**
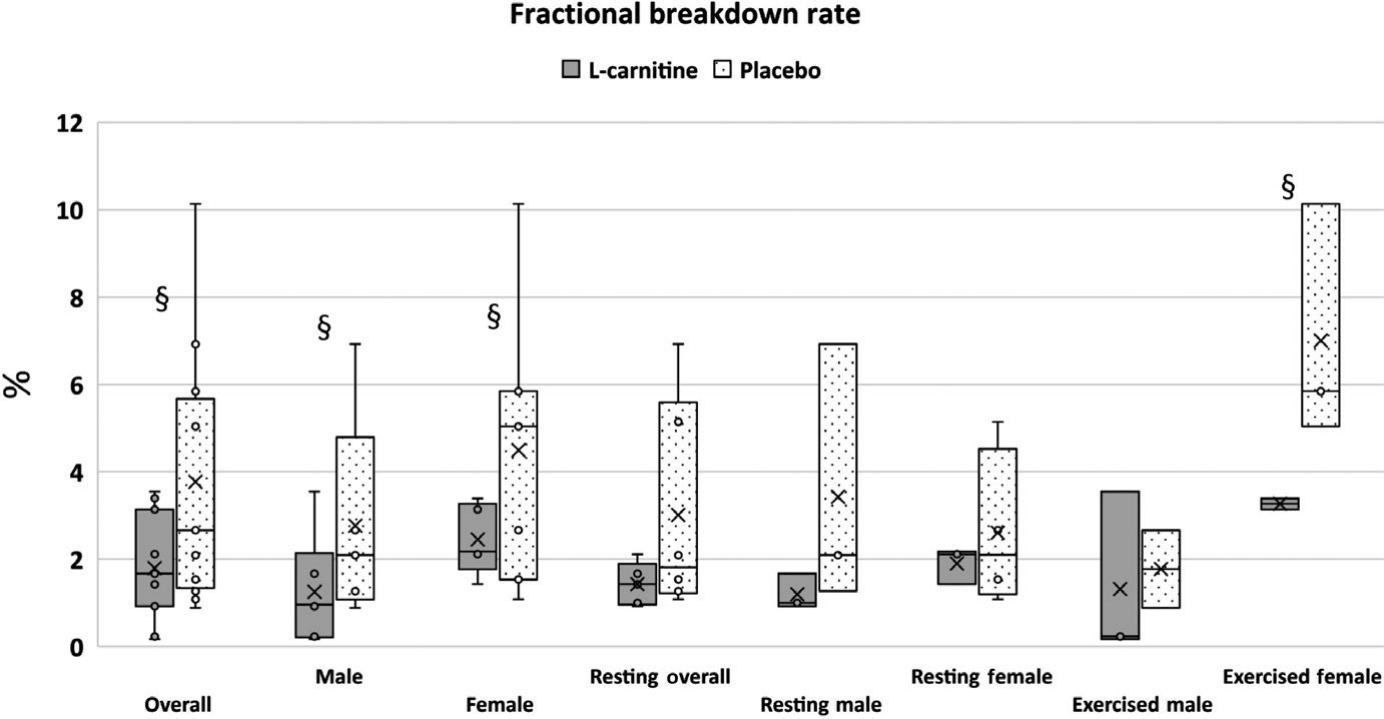
Fractional breakdown rate (%) in working Labrador Retrievers by sex and exercise status. § represents statistically significant difference between groups (*p* < .05)

Overall, the L‐carnitine group had a significantly lower FBR compared to placebo group (*p* = .043). The FBR in exercised L‐carnitine females was significantly lower than in exercised placebo females (*p* = .046; Figure 2).

There was no significance found in FSR by gender, exercise or treatment (data not reported).

### 3.3 VO_2_ and energy expenditure

**FIGURE 3:**
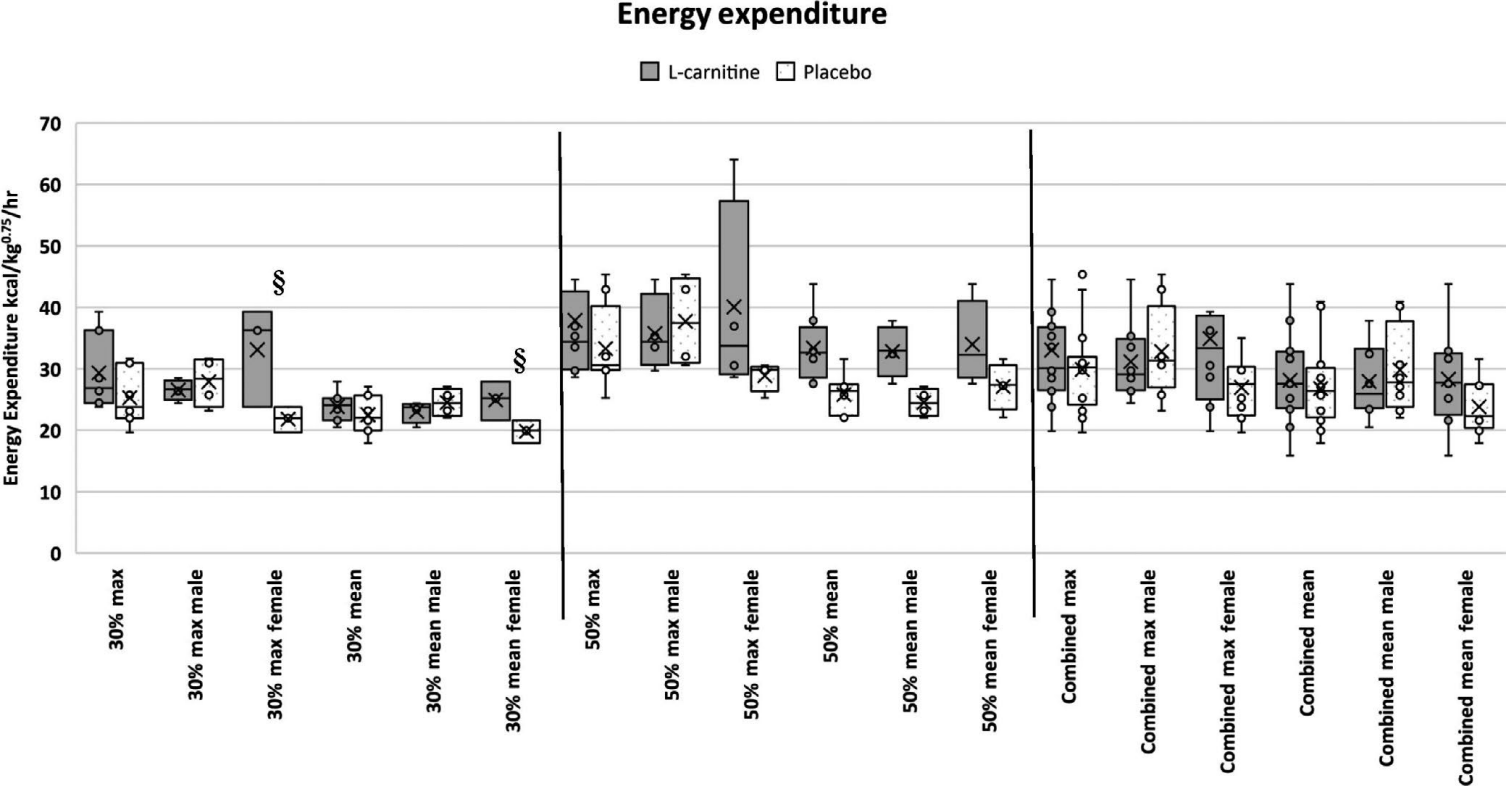
Mean and maximum energy expenditure in working Labrador Retrievers supplemented with either L‐carnitine or sucrose. § represents statistically significant difference between groups (*p* < .05)

**FIGURE 4:**
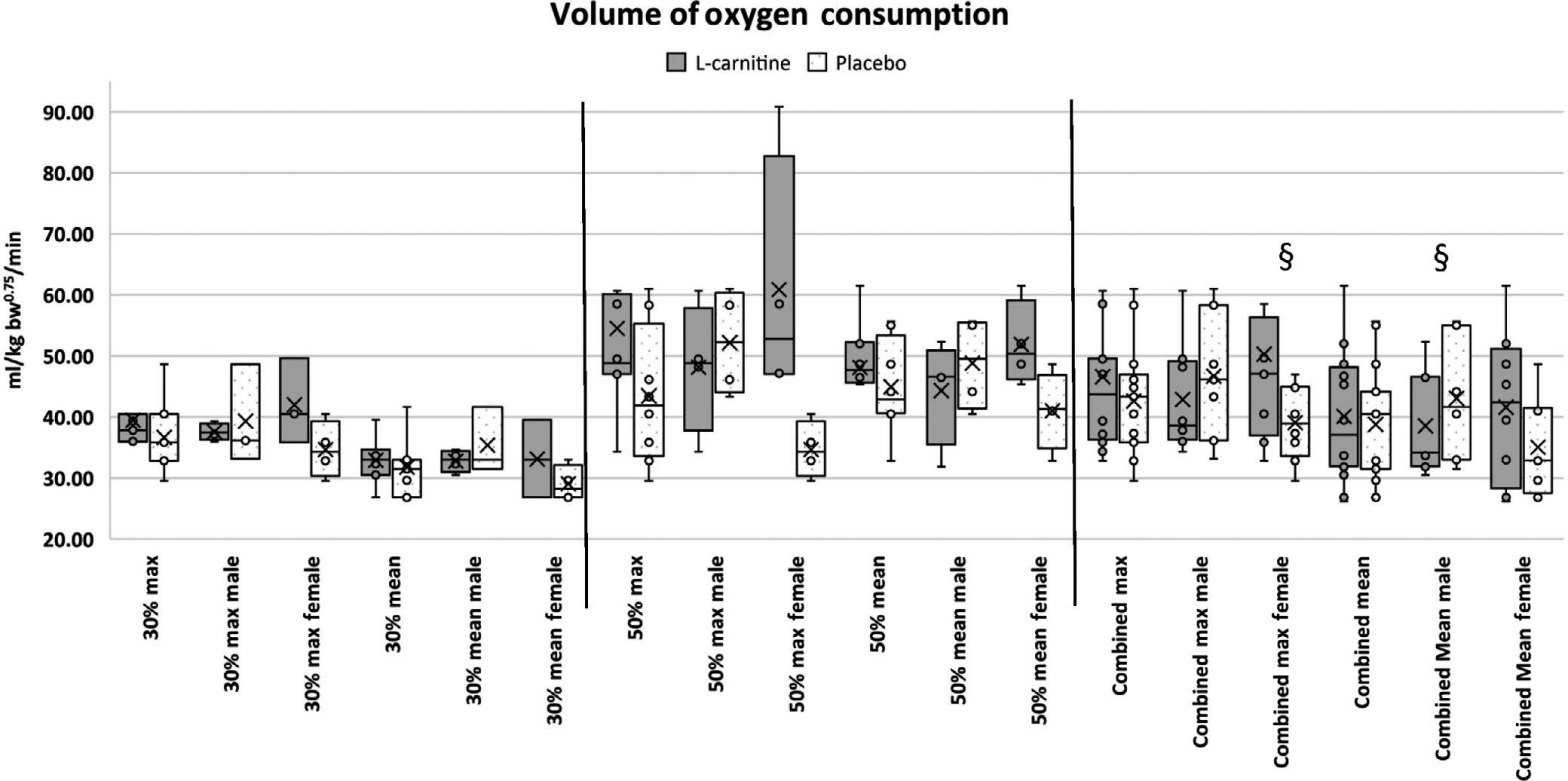
Mean and maximum energy volume of oxygen (O_2_) consumption in working Labrador Retrievers supplemented with either L‐carnitine or sucrose. § represents statistically significant difference between groups (*p* < .05)

At 30% average VO_2_ max (speed: 6.5 kph for 30 min), L‐carnitine females had a significantly higher maximum energy expenditure during exercise (EE) at 33.09 (8.19) kcal/kg bodyweight^0.75^/hr compared to placebo females at 21.8 (2.09) kcal/kg BW^0.75^/hr (*p* = .021). L‐Carnitine females also had significantly higher mean EE at 24.91 (3.15) kcal/kg BW^0.75^/hr compared to placebo females at 19.82 (1.86) kcal/kg BW^0.75^/hr (*p* = .035; Figure 3). When considering EE on a lean mass basis, L‐carnitine females had significantly higher maximum EE at 19.6 (1.30) kcal/kg lean mass/h compared to placebo females at 13.66 (4.30) kcal/kg LM/hr (*p* = .035). L‐Carnitine females also had significantly higher mean EE at 14.81 (1.74) kcal/kg LM/h compared to placebo females at 12.42 (1.18) kcal/kg LM/hr (*p* = .05). For volume of oxygen consumption (VO_2_), no significant differences were found between groups or sex. With both speeds combined, a trend was noted where L‐carnitine females had higher VO_2_ compared to placebo females (*p* = .078; Figure 4).

## 4 DISCUSSION

These studies conducted with Labrador Retrievers aimed to determine the L‐carnitine content of blood and muscle samples, to evaluate the effect of L‐carnitine on PT, and to evaluate the effect of L‐carnitine on EE and oxygen consumption.

L‐Carnitine turnover was first noted by Lennon et al. (1983) who described a loss of acylated L‐carnitine from muscle to plasma in humans after acute cycling exercise in non‐supplemented humans on a standard diet containing normal L‐carnitine content. A second study with similar design using non‐supplemented humans and cycling exercise had the same findings: a loss of acylated L‐carnitine from muscle to plasma (Hiatt, Regensteiner, Wolfel, Ruff, & Brass, 1989). Inversely, studies using L‐carnitine‐loaded human subjects saw an increase in muscle L‐carnitine content post‐exercise, indicating L‐carnitine supplemented over the standard dietary intake may influence L‐carnitine availability in the muscle (Arenas et al., 1991; Marconi, Sassi, Carpinelli, & Cerretelli, 1985). Similarly, the present study showed significantly higher free L‐carnitine in the L‐carnitine group's *biceps femoris* muscle compared to placebo group 1 hr after exercise. Although the L‐carnitine group had more esterified L‐carnitine in the muscle prior to the final run, exercise created only an 11% increase in the L‐carnitine group's L‐carnitine esters compared to a 112% increase in placebo group, indicating higher bioavailability in the L‐carnitine supplemented dogs (Table 5). Brass (2000) also described differences between human and animal studies (dog and rat) where heavy loading of L‐carnitine improved fatigue and contractile force of the muscle. It has been suggested that low availability of free L‐carnitine in the muscle may limit muscle fat oxidation during exercise (van Loon, Greenhaff, Constantin‐Teodosiu, Saris, & Wagenmakers, 2001). Orally supplementing L‐carnitine in exercised dogs increases the amount of free L‐carnitine available in the muscle, thus increasing muscle fat oxidation (Wutzke & Lorenz, 2004).

In the present study, the L‐carnitine group also had significantly higher total, free and esterified L‐carnitine content in the serum compared to the placebo group, both pre‐ and post‐endurance run. In a study using exercised human subjects, no significant difference was found in plasma concentrations of L‐carnitine in sprint runners, while endurance runners had significant plasma concentration increases in total and esterified L‐carnitine (Arenas et al., 1991). These differences were attributed to the difference in muscle fibres where endurance runners build more type 1 muscle fibre which contains more mitochondria, while sprint runners build type II muscle fibres which have fewer mitochondria. Wall et al. (2011) evaluated plasma for total L‐carnitine content immediately following cycling exercise at baseline, 12 and 24 weeks and found total L‐carnitine plasma concentration in L‐carnitine supplemented humans at 12 and 24 weeks was significantly higher compared to placebo. These findings appear to conclude that some of the discrepancies between trials may be attributed to the type of exercise performed. The present study focused on the effect and comparison of L‐carnitine and placebo supplemented from pre‐ to post‐endurance exercise and does not include baseline measurements. In future studies, baseline measurements may be useful to track the effect of L‐carnitine supplementation from pre‐supplementation throughout the trial. In this trial, L‐carnitine supplementation was effective in elevating the serum content of total, free and esterified L‐carnitine from pre‐ to post‐endurance exercise in Labrador Retrievers.

During physical exercise, biochemical processes such as hypoxia of the muscle, tissue degradation, free radical formation and sarcolemma disruption may occur (Volek et al., 2001). Degradation of tissue structures may even continue for as long as 5–10 days following eccentric exercise (Kraemer, Volek, Spiering, & Vingren, 2005). Wakshlag et al. (2002) reported English pointers and Labrador Retrievers demonstrated an increase in skeletal muscle proteolysis of the biceps femoris in peak training for the hunting season compared to same muscle proteolysis in the resting seasons. The researchers showed the peak training for the dogs caused an up‐regulation of the ubiquitinated conjugates and the p31 regulatory capping sub‐unit but the researchers did not show an up‐regulation of the catalytic core (β‐subunits) of the proteasome. In the present study, L‐carnitine was shown to help significantly lower the percentage of muscle degradation in dogs following running exercise. To date, the authors are not aware of any other studies that have examined PT in L‐carnitine supplemented canines. Agreeing with the present study, Kraemer et al. (2003) found that L‐carnitine supplementation in humans performing squat exercises reduced tissue damage by 7%–10%, allowing for increased protein synthesis at the receptor level. Keller, Couturier, Haferkamp, Most, and Eder (2013) found significantly lower muscle protein degradation in L‐carnitine supplemented rats, which was attributed to activation of the PI3/AKIt signalling pathway and inactivation of the FoxOs in the rats’ skeletal muscle. Increased levels of IGF‐1 were observed in the plasma, which has been shown to attenuate protein degradation. Conversely, Wutzke and Lorenz (2004) evaluated PT in non‐exercised humans after 10 days of L‐carnitine supplementation but found no significant difference between L‐carnitine and control groups, most likely due to the lack of exercise‐induced muscle breakdown. In the present study, half of the dogs from both treatment groups did not perform the final 24.1 km run in order to provide a resting comparison. The resting dogs from both treatment groups had a much lower FBR compared to dogs that completed the final run due to the higher rate of muscle degradation that occurs during strenuous exercise. In the exercised L‐carnitine group, skeletal muscle breakdown occurred at a significantly lower rate compared to placebo group. Saltin and Karlsson (1971) have suggested that skeletal muscle may have an increased degradation rate to supply glucose after glycogen stores are depleted during exercise. This is attributed to fat oxidation and glycogen sparing (Wall et al., 2011). Comparison groups were broken down by treatment, sex, and exercised or resting, and resulted in a small sample size per group. Increasing the number of animals in future studies may provide a more robust comparison.

In an EE study, Minikheim, Shoveller, and DiGennero (2013) found that Labrador Retrievers supplemented with L‐carnitine had higher EE compared to control but found no difference in Beagles and Miniature Dachshunds. Although no exercise was performed in Minikheim's study, the findings were consistent with the present study where Labrador Retrievers supplemented with L‐carnitine were observed to have higher EE. In the present study, supplemented female Labrador Retrievers had significantly higher mean and maximum EE at 30% VO_2_ max (Figure 3). In a study performed with humans performing cycling exercise, L‐carnitine supplemented male humans had a 6% increase in EE after 12 weeks, compared to no increase in EE in control males (Stephens et al., 2013). This was attributed to a difference in fat oxidation between L‐carnitine and control subjects. The present study observed a significant increase in oxygen consumption in L‐carnitine supplemented female dogs, similar to Vecchiet et al. (1990)’s observations of higher VO_2_ consumption in humans that were administered L‐carnitine only one time 1 hr prior to cycling exercise. Conversely, Broad, Maughan, and Galloway (2008) found no difference in VO_2_ consumption after 2 weeks of L‐carnitine supplementation in human males during cycling exercise. In the present study, oxygen consumption was not significantly different between groups or sexes. With both speeds combined, L‐carnitine females trended towards having higher VO_2_ consumption. To the authors’ current knowledge, no other studies have described a difference in the oxygen consumption and EE of male versus female subjects during L‐carnitine supplementation. This discrepancy between sexes may be due to several factors, such as hormones, heat cycles, supplementation amounts, and lack of power due to small sample size and high standard deviation. Where the present study provided a single dosage amount to all dogs regardless of weight, it may be warranted to consider dosage amount based on feed consumed, body weight or body composition. Higher body fat and lower lean mass (Table 3) in the female dogs may have resulted in higher fat oxidation rates compared to the males.

## 5 CONCLUSION

The present study concluded that Labrador Retrievers supplemented with 125 mg L‐carnitine had elevated serum L‐carnitine content, higher muscle free L‐carnitine and a lower muscle FBR determined via PT studies. Female Labrador Retrievers supplemented with L‐carnitine had higher oxygen consumption rates and EE compared to placebo female Labrador Retrievers.

## CONFLICT OF INTEREST

The authors declare no conflicts of interest.

## ANIMAL WELFARE STATEMENT

The authors confirm that the ethical policies of the journal, as noted on the journal's author guidelines page, have been adhered to and approval via the Institute of Animal Care and Use Committee under protocol FRK‐04 has been received. The authors confirm that they have followed EU standards for the protection of animals used for scientific purposes.
